# A fruit extract of *Styphnolobium japonicum* (L.) counteracts oxidative stress and mediates neuroprotection in *Caenorhabditis elegans*

**DOI:** 10.1186/s12906-023-04149-8

**Published:** 2023-09-19

**Authors:** Sara Thabit, Heba Handoussa, Nesrine S. ElSayed, Hans-Georg Breitinger, Ulrike Breitinger, Michael Wink

**Affiliations:** 1 Department of Pharmaceutical Biology, Faculty of Pharmacy and Biotechnology, German University in Cairo, New Cairo, 11835 Egypt; 2 Department of Pharmacology and Toxicology, Faculty of Pharmacy, Cairo University, Cairo, Egypt; 3 Department of Biochemistry, Faculty of Pharmacy and Biotechnology, German University in Cairo, New Cairo, Egypt; 4 Department of Biology, Institute of Pharmacy and Molecular Biotechnology, Heidelberg University, Heidelberg, Germany

**Keywords:** *Caenorhabditis elegans*, SKN-1/Nrf2, Oxidative stress, Neuroprotection, *Styphnolobium japonicum*

## Abstract

**Background:**

Despite its widespread uses in Chinese and European medicine, *Styphnolobium japonicum* (Chinese scholar tree, formerly *Sophora japonicum*) has not been extensively investigated for its potential to protect against neurodegenerative processes and to promote resistance to oxidative stress. In this study, we evaluated the neuroprotective activities of a hydroalcoholic extract from Chinese scholar tree fruits that could be possibly linked to its antioxidant properties using *Caenorhabditis elegans* as a well-established in vivo model.

**Methods:**

Survival rate in mutant *daf-16 *and *skn-1* worms, stressed by the pro-oxidant juglone and treated with the extract, was tested. Localization of the transcription factors SKN-1 and DAF-16, and expression of *gst-4* were measured. For evaluation of neuroprotective effects, formation of polyglutamine (polyQ40) clusters, α-synuclein aggregates, loss of amphid sensilla (ASH) neuronal function, and amyloid β (Aβ) accumulation (as markers for Huntington’s, Parkinson’s, and Alzheimer’s) was examined.

**Results:**

The extract, which contains substantial amounts of phenolic phytochemicals, showed an increase in the survival rate of worms challenged with juglone in *daf-16* mutants but not in *skn-1* mutants. The transcription factor SKN-1 was activated by the extract, while DAF-16 was not affected. Upon application of the extract, a significant decline in GST-4 levels, polyQ40 cluster formation, number of lost ASH sensory neurons, α-synuclein aggregation, and paralysis resulting from Aβ accumulation was observed.

**Conclusions:**

*Styphnolobium japonicum* fruit extract activated the SKN-1/Nrf2 pathway, resulting in oxidative stress resistance. It revealed promising pharmacological activities towards treatment of Huntington’s, Parkinson’s, and Alzheimer’s diseases. Polyphenolics from *Styphnolobium japonicum* may be a promising route towards treatment of CNS disorders, but need to be tested in other in vivo systems.

**Supplementary Information:**

The online version contains supplementary material available at 10.1186/s12906-023-04149-8.

## Background

*Styphnolobium japonicum* L. (SJ, Chinese scholar tree) (Fabaceae), previously known as *Sophora japonica* L., originates from East Asia. Since the tree is used as an ornamental plant, it can be found in temperate regions around the world [1, 2]. Many parts of the plant are edible after cooking including leaves, seeds, and flowers. The seed endosperm is also eaten as a dessert in Northern China by cooking with sugar [3–6].

The chemical profile of SJ shows a broad variety of secondary metabolites with a wide array of pharmacological effects. Secondary metabolites include polysaccharides and phenolic compounds such as phytoestrogens, flavonoids, which are mostly found in flowers and buds, and isoflavonoids, in fruits and seeds [7–9]. Major flavonoids and isoflavonoids that were identified include quercetin, kaempferol, genistein, and most importantly rutin, a major constituent of *Flos Sophorae Immaturus* (Flower of *Sophora japonica* L.) that is known for its antioxidant, anti-inflammatory, and anti-edemic properties [10–14].

Numerous studies describe beneficial biological effects of different SJ extracts, such as antibacterial, antihyperglycemic, anti-obesity, antihemorrhagic, cardiovascular, antitumor, anti-angiogenic, anti-atherosclerotic, antioxidant, neuroprotective, and most commonly anti-inflammatory and anti-osteoporotic activities [9, 15–19].

The antioxidant properties of an ethanol extract of SJ were manifested by its ability to prevent the production of H_2_O_2_ using a *Saccharomyces cerevisiae* yeast model [16]. Neuroprotective activities were also proposed for an extract of SJ flower buds. A previous study revealed that the extract was able to decrease the extent of neurological deficit and cerebral infarction area in a rat model of cerebral infarction induced by ischemia–reperfusion injury. These effects were attributed to the ability of SJ to decrease IL-1β expression, suppress the activation of microglia, and reduce cell apoptosis [19]. However, studies addressing the neuroprotective properties of SJ are limited.

The process of ageing, far from being fully understood, involves the gradual decline in the functionality of organisms, including the central nervous system. This is the main cause of many fatal disorders such as cancer and neurological diseases. Many neurological disorders such as Alzheimer’s disease (AD), Parkinson’s disease (PD) and Huntington’s disease (HD) have unclear pathogenesis and no treatment to reverse progress of the disease [20–22]. Thus, search of new agents that can help to prevent onset of the disease, or improve clinical symptoms is of high relevance. Different theories about ageing were suggested, such as the free radical theory, which is based upon reactive oxygen species (ROS) that are produced in the mitochondria as a by-product of metabolism. The rise in ROS levels causes oxidative stress. ROS react with proteins and nucleic acids, leading to mutations and can cause lipid peroxidation [23, 24]. Neurons can severely suffer from oxidative stress due to their high oxygen demand and low antioxidant levels [25]. Hence, agents with antioxidant activities might protect against the development of neurological disorders with age and thus preserve and enhance quality of life.

The nematode *Caenorhabditis elegans* (*C. elegans*) is an extensively used in vivo model for investigations of the molecular mechanisms by which plant extracts and compounds exert antioxidant, neuroprotective, and anti-aging effects [26, 27]. They are easy to maintain and manipulate compared to mammalian animal models and present a simple, well-defined model for a multicellular organism.

To our knowledge, the effects of SJ extracts on AD, PD and HD nematode models have not yet been tested. In this study, the in vitro antioxidant properties of a hydroalcoholic extract from dried fruits of SJ were investigated using cupric ion reducing antioxidant capacity (CUPRAC) assay. Profiling of phenolic constituents in the extract via HPLC–PDA-ESI–MS/MS had been carried out by our group previously [28]. Considering the potential role of oxidative stress in neurodegeneration, the ability to counteract oxidative stress and the participation of the transcription factors DAF-16/FOXO and SKN-1/Nrf2 in this effect were studied using different strains of *C. elegans*. A survival assay was carried out utilizing *daf-16* and *skn-1* mutant strains. In addition, assessment of DAF-16 and SKN-1 nuclear localization was performed and the expression of glutathione S-transferase-4 (GST-4) was quantified. Since protein accumulation is a characteristic feature of many CNS related disorders, the ability of SJ extract to counteract this effect was tested using special models of *C. elegans*. Quantification of polyglutamine (PolyQ40) clusters, a pathological feature in many diseases like HD, was assessed in strain AM141, and α-synuclein aggregate accumulation, implicated in PD, in strain NL5901. Preserving functional amphid sensilla (ASH) neurons was studied using HA759 strain. Finally, the influence of the extract on amyloid β (Aβ) aggregation characterizing AD was evaluated through studying paralysis in worms expressing human Aβ genes in their muscles [29, 30].

## Methods

### Plant extraction

Dried fruits of SJ were commercially obtained from a company specializing in medicinal plants, Kräuter Schulte, Germany. There are no legal, ethical or other restrictions that would limit the use of this material. A voucher specimen was deposited at Pharmaceutical Biology Department Herbarium, German University in Cairo, Egypt (Specimen no. 0047).

A hydroalcoholic extract of 1 kg dried fruits was prepared by grinding them, followed by three rounds of overnight maceration at 55 °C in 2.5 L of 70% methanol with agitation. The extract was filtered, concentrated in vacuum, at 37 °C with a rotavap, and lyophilized. The crude extract was stored at -20 °C. Profiling of phenolic constituents in the extract was performed via HPLC–PDA-ESI–MS/MS as previously described [28].

### CUPRAC assay

This assay was carried out to assess in vitro antioxidant capacity by estimating the ability of SJ extract to reduce cupric (Cu^2+^) to cuprous (Cu^+^) ions [30]. Butylated hydroxyl anisole (BHA) was utilized as a standard (Sigma-Aldrich, Darmstadt, Germany), at concentrations of 50, 100, 200, and 400 µg/mL. One mL of 7.5 mM neocuproine (Sigma-Aldrich, Darmstadt, Germany), dissolved in ethanol, 1 mL of 0.01 M CuCl_2_, and 1 mL of 1 M NH_4_Ac were added to 0.3 mL of either the standard or SJ extract (50, 100, 200, and 400 µg/mL). Purified water was added to all test tubes to yield 4.1 mL final volume. Absorbance of the mixture was read at 450 nm versus a blank after 30 min. Data points were plotted and fitted to a linear regression using the equation y = ax + b; EC_50_ for SJ extract was calculated using the equation: EC_50_ = 0.5-b/a.

### Strains of *Caenorhabditis elegans*

*C. elegans* strains employed in the various assays, in addition to *E. coli* OP50, were obtained from Caenorhabditis Genetics Center, Minnesota University, USA. The strains utilized were CF1038 (*daf-16(mu86)I*), EU1 [*skn-1(zu67) IV/nT1(IV;V*)], TJ356 (*zIs356[daf-16p::daf-16a/b::GFP* + *rol-6*]), LD1 [*skn-1b/c::GFP* + *rol-6(su1006)*], CL2166 (*dvIs19[pAF15(gst-4::GFP::NLS)*]), AM141 (*rmls133[unc-54p::Q40::YFP]*), HA759 *(rtIs11[osm-10p::GFP* + *osm10p::HtnQ150* + *Dpy-20(* +*)])*, NL5901 (*Punc-54::alpha-synuclein::YFP* + *unc-119*), CL4176 (*dvIs27[myo-3p::A-Beta (1–42)::let-851 30UTR*) *C rol-6(su1006)*]) X, and CL802 [*smg-1(cc546) I; rol-6(su1006) II*]. Plates containing nematode growth medium supplied with *E. coli* OP50 as food for worms were incubated at 20 °C incubator during experiment time. CL4176 and CL802 strains only were kept at 16 °C incubator. To obtain worms with synchronized age, we followed a previously established protocol of bleaching adult hermaphrodites to take their eggs using 5 M NaOH and 5% NaOCl in a ratio of 1:3 [30].

### Survival assay using juglone

Age-synchronized nematodes CF1038 (*daf-16* mutant nematodes) and EU1 (*skn-1* mutants) at L1 stage were divided into eight groups (70–80 worms each) and grown using S-medium and *E. coli* OP50 at 20 °C. All groups were left for 48 h after treatment. The hydroalcoholic SJ extract (at concentrations of 100, 200, and 300 µg/mL) was given to three groups.

( −)- Epigallocatechin gallate (EGCG) (≥ 95%, Sigma-Aldrich, Darmstadt, Germany) at 50 µg/mL was used as a positive control. Three negative control groups were also used, two groups devoid of treatment and a solvent control group treated with methanol (2.1% final concentration). One group of nematodes received 100 μg/mL of rutin. After 48 h, all groups received a lethal dose (80 µM) of juglone (Sigma-Aldrich GmbH, Darmstadt, Germany), followed by another 24 h of incubation (except for one of the untreated control groups). Finally, dead and alive worms were counted and recorded. Worms were assumed to be dead when they could not be provoked upon stimulation with a wire made of platinum. Four independent repetitions of the experiment were performed and data calculated as mean ± SEM of the survival rate percentage. Results were compared using GraphPad prism (5.01) and significance tested using one-way ANOVA followed by Bonferroni’s method (post hoc) [28].

### Determining localization of the transcription factor DAF-16

The *C. elegans* strain TJ356, having *daf-16* fused to GFP, was utilized for observing the localization of DAF-16 transcription factor. L1 synchronized larvae were divided into seven groups (30–50 worms each) and treated as described above using S-medium + *E. coli* OP50 at 20 °C. TJ356 worms were investigated after 24 h using fluorescence microscopy. Worms were fixed on glass slides using 10 mM sodium azide for paralysis. A BIOREVO BZ-9000 microscope, equipped with a mercury lamp, at an excitation and emission wavelengths of λ_ex_ 480/20 nm and λ_em_ 510/38 nm (Keyence Deutschland GmbH, Neu-Isenburg, Germany) and an Axiostar Plus 37081 fluorescence microscope (Carl Zeiss) were used to view the nematodes. Pictures of 30–40 worms were randomly viewed for analysis using 20X objective lens and a constant time of exposure. Worms were counted after dividing them according to the localization of DAF-16::GFP to cytosolic, nuclear, or intermediate localization. Analysis and comparing nuclear results percentage between different groups were done as described above. Three independent sets of the experiment were performed and averaged.

### Determining localization of the transcription factor SKN-1

The *C. elegans* strain LD1, having *skn-1* coupled to GFP, was used to determine localization of SKN-1 transcription factor. L1 nematodes were divided to six groups (20–30 worms each). All groups were kept for 48 h in S-medium supplied with *E. coli* OP50 at 20 °C, and visualized afterwards with fluorescent microscope. Approximately 20 worms were visualized and analyzed at constant exposure time using 20X magnification. Three independent assays were run and worms were divided and counted according to SKN-1::GFP localization as cytosolic or nuclear. Percentage of worms demonstrating nuclear localization was compared as described above.

### GST-4 quantification

The CL2166 strain of *C. elegans*, with *gst-4* fused to GFP, was used in this assay. Nematodes at the L1 stage of development were divided to seven groups (20–30 worms each) and then left to grow for 48 h in S-medium and *E. coli* OP50 at 20 °C. Imaging of 20–30 worms was done using constant exposure time and 10X magnification. The assay was performed three different times, and mean fluorescence intensity for the entire body of the nematodes was measured. Analysis was done using ImageJ version 1.48 (NIH, Bethesda, MD, USA). Data comparison was performed as described formerly.

### Quantitative estimation of polyQ40 clusters

AM141 transgenic nematodes, with polyQ40 coupled to YFP, were age synchronized and used at the L1 stage. Experimental groups were set up to eight groups, each having 30–40 worms, and left for 72 h in S-medium + *E. coli* OP50 at 20 °C. Worms were viewed with fluorescent microscopy. Around thirty worms were pictured via 10X lens with constant exposure time. Testing was carried out three times, and the number of PolyQ40::YFP clusters, found within muscles, was scored all over the bodies of worms. Results are shown as mean number of clusters ± SEM. Comparison of results was done as previously described.

### Chemotaxis assay

HA759 transgenic nematodes were used to assess the viability of ASH neurons in the different treatment groups based on the chemotaxis behavior of worms as described before [31]. Age synchronized L1 stage worms were sorted into six groups, 100–150 worms each, and kept at 20 °C in S-medium containing *E. coli* OP50 for 72 h. Chemotaxis 60 mm NGM plates were divided into two halves and sodium azide (0.5 M, 1.5 μL) and 0.1% benzaldehyde dissolved in 99.8% ethanol (1.5 μL) were put to the attractant zone. The control zone on the opposite side of the plate contained 1.5 μL of 0.5 M sodium azide and 1.5 μL of 99.8% ethanol. Afterwards, the worms were washed using M9 buffer thrice to get rid of *E. coli* and around 100 worms (5 μL) were collected from each group and put in the middle of the plates. All plates were incubated at 20 °C for 1.5 h and the number of worms on each side was counted afterwards. Chemotaxis index was calculated using the following equation: (number of worms in the attractant zone – number of worms in the control zone) / total number of worms. The data of three assays was included and expressed as mean ± SEM. Data comparison was performed as mentioned previously.

### Estimation of α-synuclein accumulation

NL5901 transgenic strain was utilized for the assessment of α-synuclein aggregate accumulation. This strain resembles PD by having human α-synuclein linked to YFP expressed in the muscle cells of nematodes. Age-synchronized worms were categorized into six groups, 30–40 worms each, and left for 96 h in S-medium with *E. coli* OP50 at 20 °C. The nematodes were observed using fluorescence microscopy as described formerly using 10X lens and constant exposure time. Three independent experiments were carried out, and an average of 15–20 worms was pictured in each group. Intensity of fluorescence was measured in the head and pharynx area. Quantification was done using ImageJ, and results were compared as previously described. Results are shown as mean ± SEM of relative fluorescence intensity.

### Paralysis test

This assay uses transgenic CL4176 nematodes. They carry human Aβ gene in their muscles and exhibit paralysis as a result of toxicity from Aβ aggregation. The control strain CL802, devoid of Aβ gene, was also incorporated in this assay to exclude any effect arising other than that of Aβ expression. CL4176 worms were sorted into six groups of 60–80 worms each. SJ extract was used in two concentrations, 300 and 500 µg/mL. EGCG and rutin groups were given at doses of 100 and 300 µg/mL, respectively. The assay was done and analyzed as previously described [30]. Treatment was done on NGM plates and they were kept at 16 °C for duration of 24 h then *E. coli* OP50 was added to the middle of the plates and they were kept at 16 °C for another 24 h. Afterwards, bleaching was done to get age-synchronized worms and about 60–80 eggs were added to the center of each plate.

NGM plates treated with worms were left for 48 h at 16 °C, followed by temperature upshift to 25 °C for stimulating Aβ gene expression. Twenty-two hours later, scoring was done for the worms every 2 h for a period of 12 successive hours. Paralyzed worms are considered the ones that could not be provoked after touching with a platinum wire or only moved their heads. Data of three different runs were averaged and presented as mean ± SEM, and PT_50_ was obtained using Kaplan–Meier method of survival analysis. Comparison of results was performed via one-way ANOVA and Bonferroni’s test.

## Results

The in vitro estimation of the antioxidant capacity of the SJ extract was carried out via the CUPRAC test. Active antioxidants in the extract reduced the CUPRAC reagent producing an orange color whose absorbance was read at 450 nm. The positive control, BHA, showed an EC_50_ of 167 ± 0.5 µg/mL, while SJ extract showed a value of 498.3 ± 0.1 µg/mL.

**Fig. 1:**
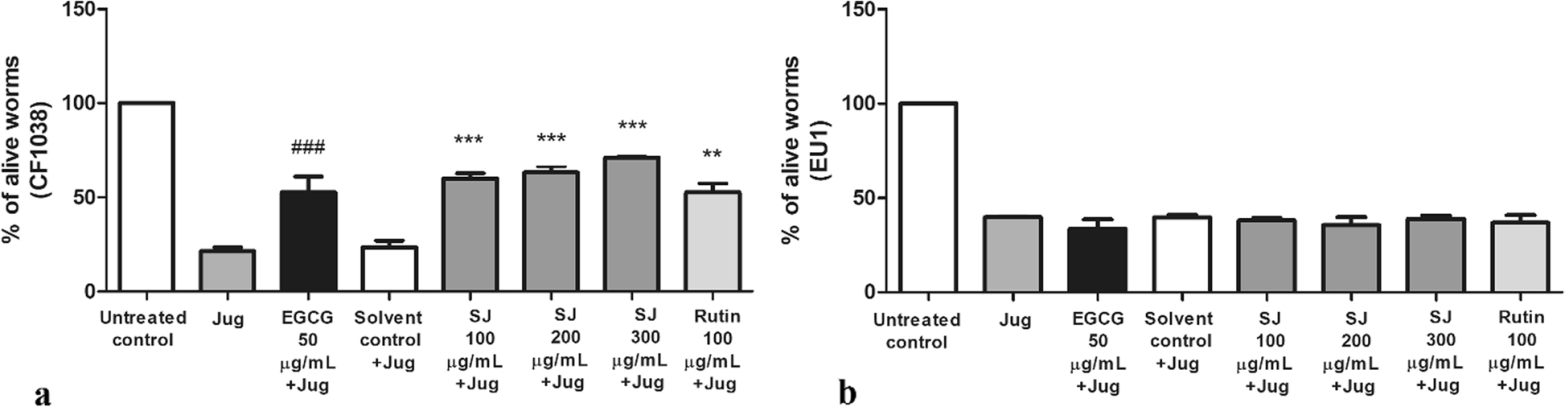
Survival rates of *C. elegans* mutants (pretreated with different treatments) stressed with 80 µM juglone. **a** CF1038 mutants. **b** EU1 mutants. Data is presented as mean survival value ± SEM for four assays. ###*p* < 0.001 in relation to juglone group. **p < 0.01 and ***p < 0.001 in relation to solvent control + juglone. The error bars in untreated controls and juglone treated group are too small to be resolved

In a survival assay with *C. elegans* (CF1038 mutant), challenged by juglone (80 µM) for 24 h, worms that were pretreated with SJ extract showed significantly higher survival rates at all tested doses in comparison to worms treated with solvent control + juglone. The protective effect was similar to that of the known antioxidant polyphenol EGCG from green tea, used as a positive control. SJ at 300 µg/mL produced the highest survival rate of 71 ± 1% (*p* < 0.001). Although the survival rate in the rutin group was lower than that of SJ extract, it still was a significant increase of 52.6 ± 4.7% (*p* < 0.01) over solvent control (Fig. 1a). In contrast, all treated groups of SKN1- deficient EU1 mutant nematodes ([*skn-1(zu67) IV/nT1(IV;V*)]) did not show any significant difference in survival rates compared to their respective negative controls (Fig. 1b), indicating the importance of the transcription factor SKN1.

**Fig. 2:**
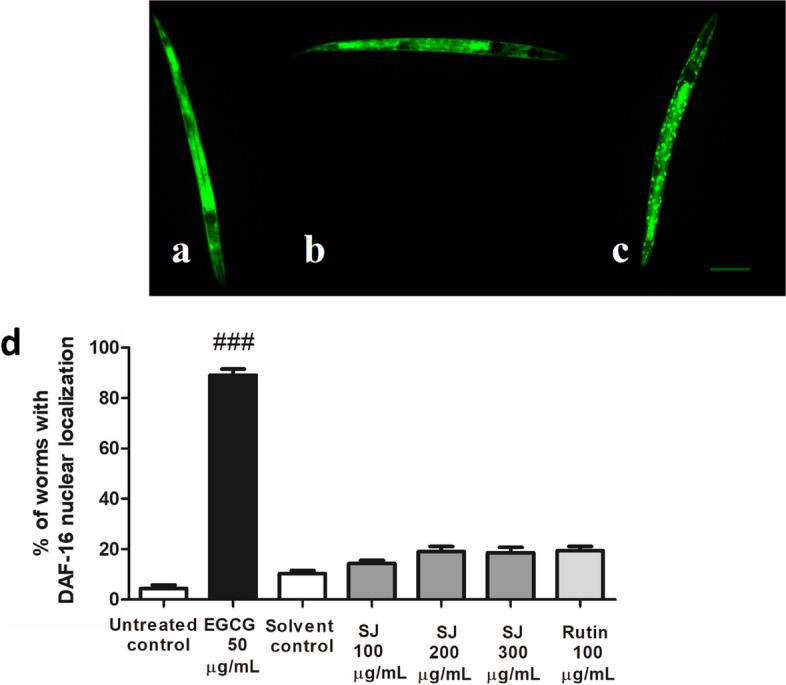
DAF-16 translocation in *C. elegans* worms. **a** Cytosolic. **b** Intermediate. **c** Nuclear. Scale bar = 100 µm. **d** Percentage of worms exhibiting nuclear localization. Values are expressed as the mean ± SEM of three tests. ###*p* < 0.001 is the difference in comparison to the untreated control

The relocation of the transcription factor DAF-16 from cytosol to the nucleus was not affected by SJ extracts. SJ extract-treated groups did not display a significant difference in DAF-16 nuclear localization compared to solvent control. Similarly, DAF-16 localization in rutin-treated animals was not significantly different from control (Fig. 2).

**Fig. 3:**
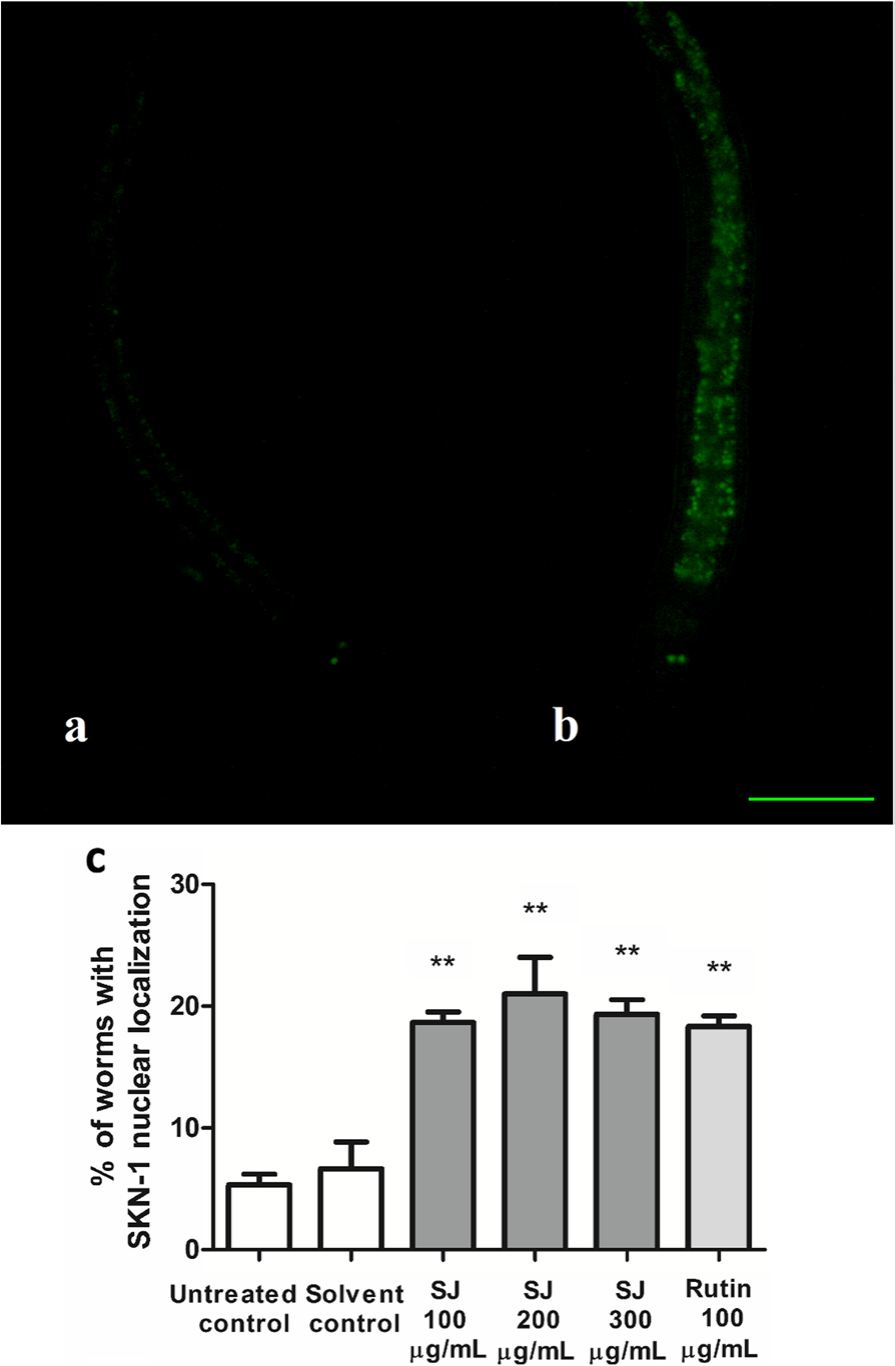
SKN-1 translocation in *C. elegans*. **a** Cytosolic localization. **b** Nuclear localization. Scale bar = 200 µm. **c** Percentage of nematodes with nuclear localization. SJ treatment leads to a remarkable nuclear localization of SKN-1. Values are expressed as mean ± SEM of three tests. **p < 0.01 is given in comparison to solvent control

The LD1 transgenic *C. elegans* strain, where the *skn-1* promoter is conjugated to GFP, was used to test the involvement of the SKN-1/Nrf2 pathway in extract activity. SJ extract, at all used doses, led to a remarkable nuclear translocation of SKN-1 in relation to solvent control. SJ 200 µg/mL revealed the highest percentage of nuclear localization (21%) compared to solvent control (6%). Changes in relocation were also significant in the rutin group, at 18.3% (*p* < 0.01) (Fig. 3).

**Fig. 4:**
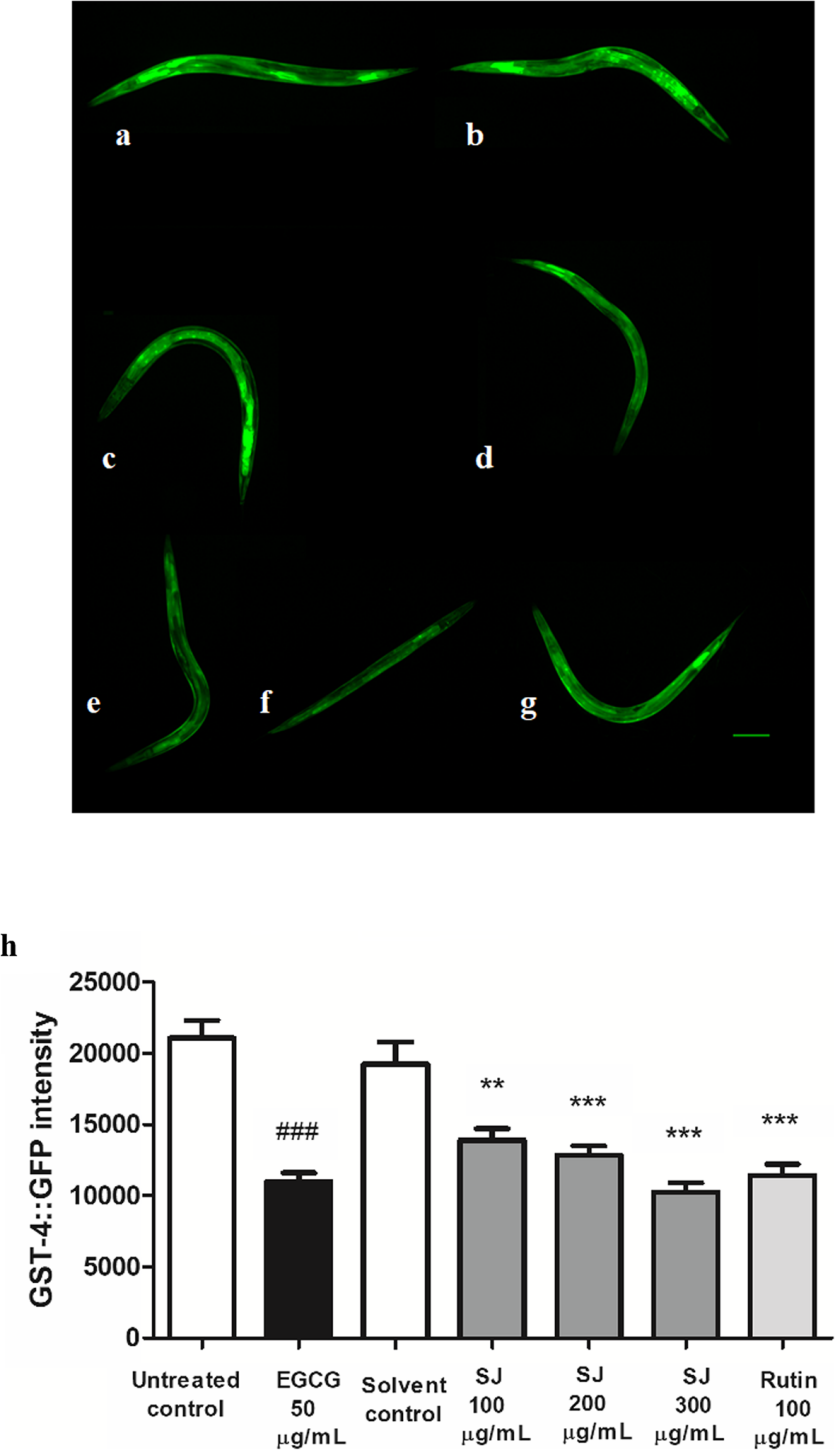
Expression of GST-4 in *C. elegans*. **a** Untreated control. **b** Solvent control. **c** SJ 100 µg/mL. **d** SJ 200 µg/mL. **e** SJ 300 µg/mL. **f** EGCG 50 µg/mL. **g** Rutin 100 µg/mL. Scale bar = 100 µm. (h) SJ extract decreases GST-4 levels in mutant CL2166 worms relative to solvent control. Results are expressed as mean ± SEM of three different runs. ###*p* < 0.001 is the difference in comparison to untreated control. ***p < 0.001 and **p < 0.01 in comparison to solvent control. Photos from three independent experiments are provided as Supplementary Fig. S1 (Additional file 1)

*Gst-4* is a downstream target gene for the SKN-1/Nrf2 transcription factor, responsible for mediating longevity and oxidative stress resistance in *C. elegans*. It contributes to the oxidative stress response as a part of Phase ӀӀ detoxification [32]. A dose-dependent decline in fluorescence intensity was noticed upon treatment with different doses of SJ extract in relation to that of the solvent control. SJ at a concentration of 300 μg/mL revealed the highest decline in fluorescence intensity, 47%, compared to solvent control. This result is comparable to EGCG positive control, showing 48% decrease in fluorescence in comparison to untreated control. The rutin treated group also significantly reduced fluorescence levels by 41% (*p* < 0.001) (Fig. 4).

**Fig. 5:**
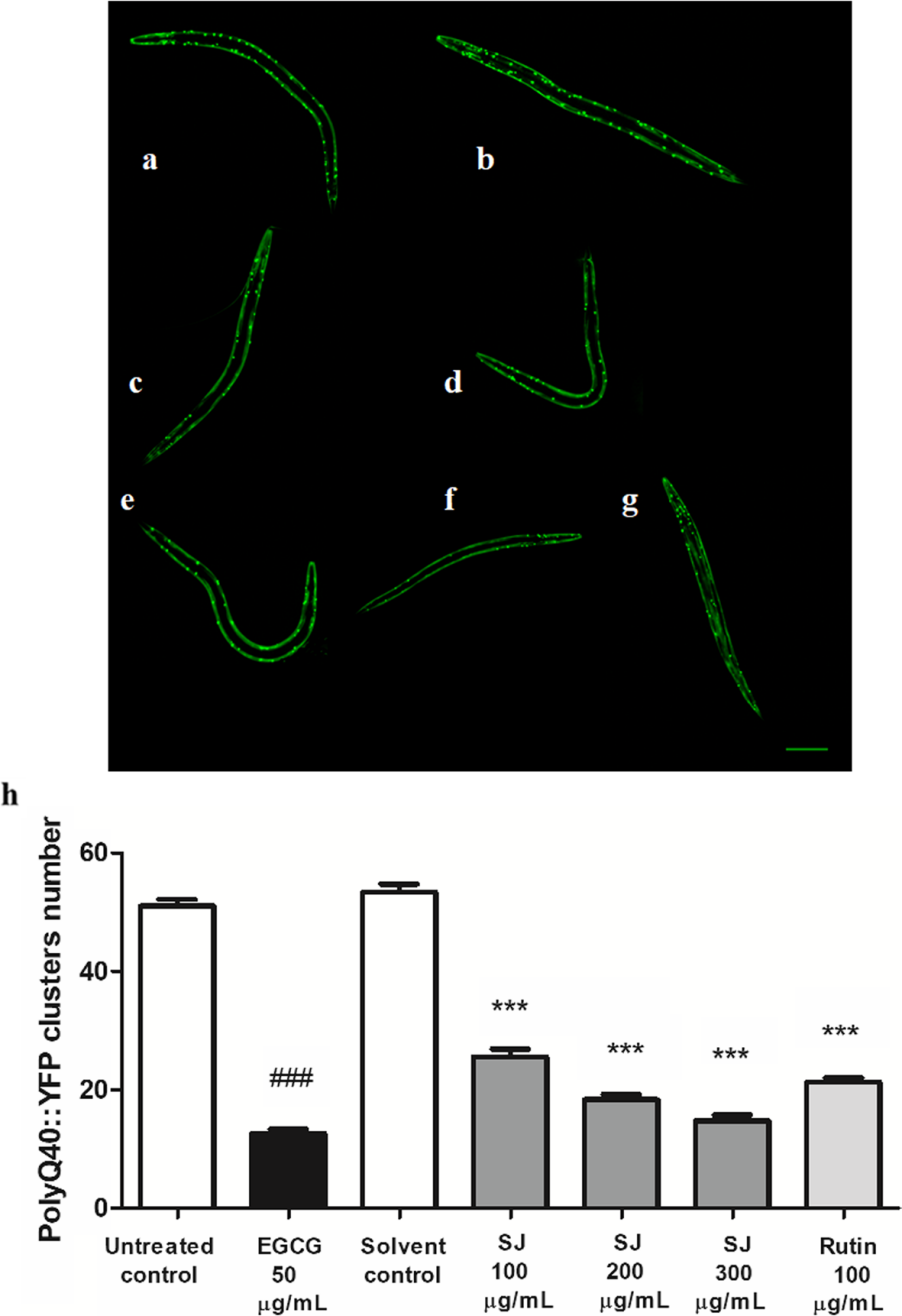
Micrographs of worms showing polyQ40 clusters. **a** Untreated control. **b** Solvent control. **c** SJ 100 µg/mL. **d** SJ 200 µg/mL. **e** SJ 300 µg/mL. **f** EGCG 50 µg/mL. **g** Rutin 100 µg/mL. Scale bar = 100 µm. (h) SJ extract decreases polyQ40::YFP clusters in mutant AM141 worms relative to solvent control. Data is expressed as mean ± SEM of three independent assays. ### p < 0.001 in comparison to the untreated control and ***p < 0.001 in comparison to solvent control. Photos from three independent experiments are provided as Supplementary Fig. S2 (Additional file 2)

To test for neuroprotective activities of SJ, polyQ40 cluster formation was investigated. The number of polyQ40 clusters, corresponding to neuronal damage, was substantially reduced upon treatment with SJ extract. The SJ extract, 300 µg/mL, showed a 72 ± 1% reduction in cluster numbers in comparison to solvent control. The positive control EGCG group demonstrated a similar value of 75 ± 1% reduction. The rutin control group also revealed a significant decline of 60 ± 1% (*p* < 0.001) (Fig. 5).

**Fig. 6:**
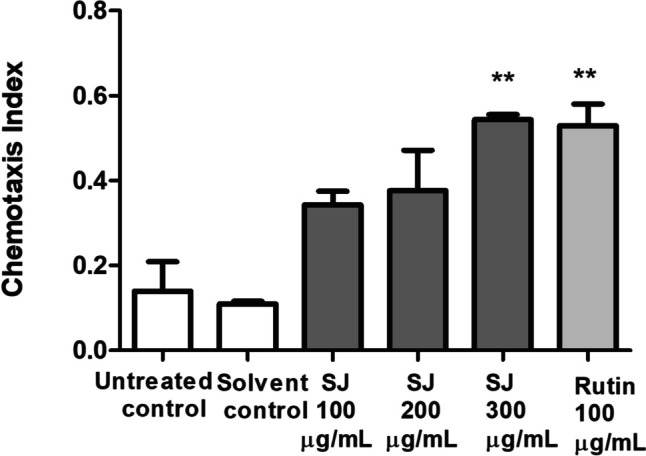
Chemotaxis assay utilizing HA759 nematodes. SJ extract 300 µg/mL increases chemotaxis index compared to solvent control group. Results are presented as mean ± SEM of three assays. **p < 0.01 versus solvent control

PolyQ formation also affects the function of ASH neurons leading to deficiency in the normal chemotactic behavior of worms towards odors like that of benzaldehyde. HA759 worms treated with SJ extract at 300 µg/mL showed high chemotaxis index of 0.54 ± 0.01 compared to the solvent control group, having index of 0.11 ± 0.01. The rutin group expressed also a significantly increased chemotaxis index of 0.53 ± 0.05 (*p* < 0.01) (Fig. 6).

**Fig. 7:**
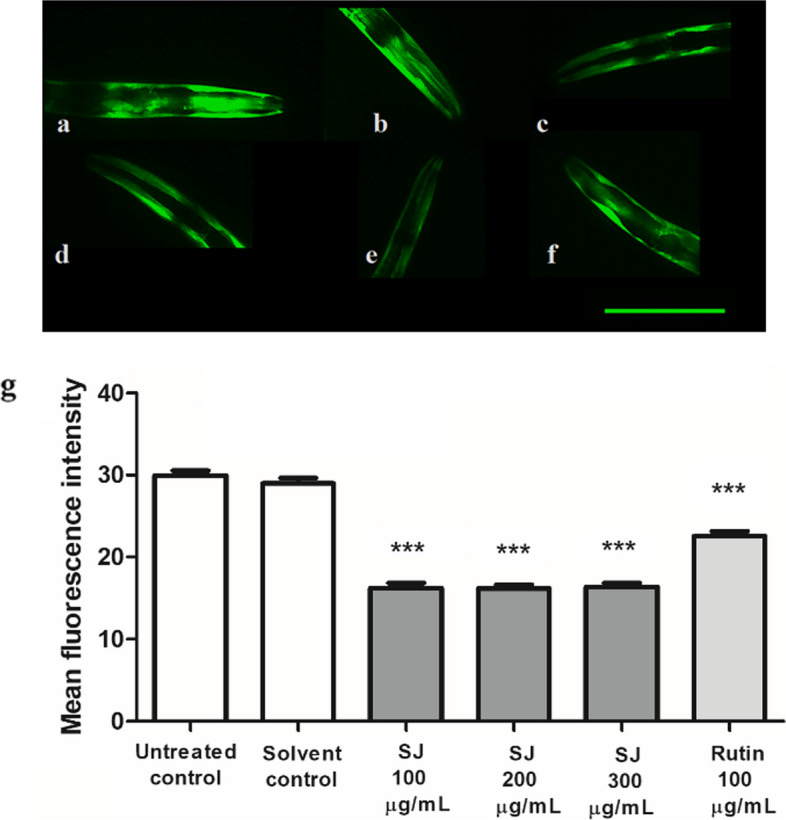
Effect of SJ extract on α-synuclein accumulation. **a** Untreated control. **b** Solvent control. **c** SJ 100 µg/mL. **d** SJ 200 µg/mL. **e** SJ300 µg/mL. **f** Rutin 100 µg/mL. Scale bar = 50 µm. **g** SJ extract decreases α-synuclein accumulation in NL5901 worms in relation to the solvent control group. Results denote mean ± SEM of three runs. ***p < 0.001 in comparison to solvent control

To characterize the neuroprotective properties of SJ extract more closely, quantification of fluorescence intensity representing α-synuclein accumulation was performed. SJ treatment significantly decreased α-synuclein accumulation, a decline of 43% ± 0.4% relative to the control was observed for 300 µg/mL of SJ extract. The rutin control exhibited a significant reduction of 22 ± 0.5% (*p* < 0.001) (Fig. 7).

**Fig. 8:**
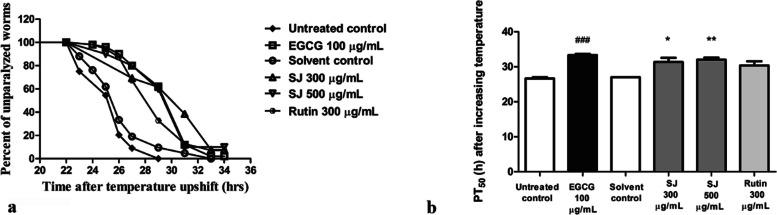
Paralysis assay using CL4176 worms. **a** Effect of SJ extracts on Aβ-induced paralysis. SJ treatment caused a delay in time of paralysis compared to the solvent control group. **b** PT50 values for different treatments, data is shown as mean ± SEM of three assays. *p < 0.05 and **p < 0.01 compared to the solvent control group and ###*p* < 0.001 compared to untreated control group. The error bar in the solvent control group is too small to be resolved

For further delineation of neuroprotective activities of SJ, a paralysis assay was conducted using temperature-sensitive CL4176 worms. When the temperature rises, human Aβ peptide accumulates in the muscles of the nematodes, and they become paralyzed [33]. PT_50_, the median time when 50% of worms are paralyzed, was determined. Both SJ 300 µg/mL and SJ 500 µg/mL revealed a significant delay in PT_50_ of 4.0 ± 1.2 h (*p* < 0.05) and 5.0 ± 0.5 h (*p* < 0.01), respectively compared to solvent control. 300 µg/mL rutin revealed a non-significant 3.0 ± 1.2 h delay, while the positive control EGCG 100 µg/mL showed a highly significant 6.6 ± 0.3 h delay compared to untreated control (*p* < 0.001) (Fig. 8). Worms that did not express Aβ (control strain) were not paralyzed upon exposure to any of the treatments.

## Discussion

Aging is a normal physiological process that is accompanied by problems in protein homeostasis. A substantial decrease in the rate of protein synthesis and concomitant loss of the ability to degrade misfolded proteins occurs resulting in their accumulation leading to various proteinopathies like Huntington’s disease, Parkinson’s disease, and Alzheimer’s disease [34]. Clinical studies revealed that CNS diseases like AD and PD lead to oxidative stress and damage to DNA, lipids, and proteins of the CNS [35, 36]. Indeed, the degree of oxidative stress is directly correlated to the process of aging and development of neurodegenerative disorders. Therefore, antioxidants are expected to protect against and decrease the progression of neurodegenerative diseases.

Plants rich in phenolic metabolites have previously shown the ability to interfere with protein folding and reduce or prevent their aggregation [26, 37].

We have previously reported the phytochemical characterization of SJ hydroalcoholic extract and identified numerous phenolic compounds including kaempferol, quercetin, and apigenin. The extract revealed promising antioxidant activity and conferred resistance to oxidative stress in *C. elegans* and mouse models, making it a good candidate as a potential neuroprotective agent [28]. In the present study, CUPRAC reduction assay results demonstrate that the antioxidant activity of the SJ extract is not mediated by a direct radical scavenging effect. However, in vivo antioxidant effects depend on other factors like bioavailability, trafficking inside different organisms, and triggering of biological mechanisms such as modulation of stress response genes affecting antioxidant enzymes levels.

Rutin, a flavone, is known to exert several modes of antioxidant action including elevation of enzymatic and non-enzymatic antioxidant levels, free radical scavenging, and chelation of metal ions [38]. A rutin control was performed in all assays to examine the effect of the total extract in comparison to a single, well-characterized, polyphenolic compound.

Longevity and stress resistance in *C. elegans* are mediated through two main transcription factors, DAF-16 and SKN-1 [39]. We have previously shown that SJ extract exhibits antioxidant and oxidative stress-reducing activities in wild type worms as demonstrated by significantly increased survival rates, for nematodes exposed to a lethal dose of juglone, and a concomitant decrease of basal ROS levels; in addition to enhanced superoxide dismutase-3 (SOD-3) levels [28]. To explore the involvement of DAF-16/FOXO and SKN-1/Nrf2 pathways, two mutant strains were used for survival assays: (i) the mutant *daf-16* strain, CF1038, and (ii) a mutant *skn-1* strain, EU1. Both strains were used for assaying survival after exposure to oxidative stress upon exposure to juglone.

DAF-16 translocation was measured to assess the contribution of the transcription factor DAF-16 to SJ extract activity. Normally, DAF-16 is found in an inactive form in the cytosol where fluorescence is seen distributed all over the body of worms. DAF-16 activation leads to its translocation to the nucleus, visible by punctate fluorescence. Activation and translocation of DAF-16/FOXO are triggered by different stressors such as heat and oxidative stress. This activation contributes to the modulation of subsequent stress response genes and genes associated with metabolism and life span regulation [40].

The transcription factor SKN-1 of *C. elegans* is orthologous to mammalian Nrf2. This transcription factor contributes to numerous regulatory pathways responsible for stress resistance and life span extension. Stressful conditions or xenobiotics lead to its accumulation in nuclei of the intestine with subsequent activation of downstream genes related to Phase ӀӀ detoxification [41, 42].

Survival assays using *daf-16* mutants revealed an increase in survival rate in worms pretreated with SJ extract, demonstrating that the activity of the extract is independent of the DAF-16 transcription factor. In contrast, survival rates of *skn-1* mutants were not changed by the absence or presence of SJ extract or rutin. This indicates the importance of SKN-1in the activity of both, SJ extract and rutin. Furthermore, SJ extract at all doses, as well as the rutin control did not produce any significant induction of DAF-16 nuclear localization, indicating the absence of this transcription factor in their action. A similar observation has been reported previously for rutin [43]. In contrast, both, SJ and rutin, strongly and significantly induced nuclear translocation of *skn-1* proving its involvement in their mechanism of action. Phytochemicals like diallyl trisulfide from garlic and baicalein from Baical skullcap have been shown to lead to activation of the SKN-1 pathway but not of DAF-16. This in turn leads to lifespan-extending effects and modulation of stress resistance in *C. elegans* [44, 45].

Extracts of plants like *Ginkgo biloba* and *Cassia fistula* have previously shown the ability to down-regulate the expression of genes as *gst-4* leading to oxidative stress resistance properties, an effect that could be mediated through SKN-1 [30, 46]. Our observation that SJ extract significantly reduced *gst-4* expression is consistent with these previous reports, emphasizing the contribution of SKN-1-mediated pathways to the mechanism of action of SJ extract, and rutin [43].

Notably, the SKN-1 pathway is believed to protect the worms from oxidative stress-related protein damage [41]. Based on its noted antioxidant activity, the extract was tested for its neuroprotective properties. Glutamine duplications are associated with Huntington’s disease, as well as other neurodegenerative diseases, resulting in mitochondrial impairment and dysfunction [47]. A PolyQ40 assay revealed notable decrease in aggregate number in the extract-treated groups compared to solvent control, consistent with potential neuroprotective effects. Similar effects were observed for treatment of nematodes with rutin.

For further testing of the neuroprotective effect of the extract, a chemotaxis assay was performed using the *C. elegans* HA759 strain expressing Htt-Q150 within ASH neurons. Htt-Q150 is a human huntingtin protein fragment characterized by 150 repeats of CAG trinucleotide leading to the expansion of PolyQ tract and loss of ASH neuronal function [48]. Live ASH neurons mediate nematodes’ chemosensory behavior towards different stimuli such as odorants. PolyQ expression results in problems in chemotaxis ability due to death of ASH neurons in *C. elegans* worms [49]. Treatment with SJ extract resulted in a significant increase in chemotactic ability of worms compared to the vehicle control group. Rutin showed similar effects. This result confirms the ability of the extract to protect against formation of PolyQ aggregates as found in HD, and this effect may be due to activation of SKN-1, although other mechanisms cannot be excluded from our experiments. A previous study has also highlighted the importance of the contribution of SKN-1 pathway in protection from toxicity caused by polyglutamine [31].

Oxidative stress has always been linked to nigral loss of dopaminergic neurons in the brains of Parkinson’s disease patients [50]. Cells try to counteract the damaging effect of oxidants to preserve the redox balance through activation of several antioxidant defense pathways. Upon activation of SKN-1 and its nuclear translocation, it binds to antioxidant genes through the antioxidant responsive element leading to oxidative stress resistance and preservation of dopaminergic neurons [42, 51].

The formation of α-synuclein aggregates is considered an important factor in the pathogenesis of several Lewy body disorders, such as Parkinson’s disease, multiple system atrophy, and others. Oxidative stress was associated with the formation of fibrils, eventually leading to the development of α-synuclein aggregates [52]. Here, we have shown that SJ extract was able to decrease this formation of α-synuclein aggregates. This effect could be linked to constituents in the extract including kaempferol, whose anti-fibrillogenic and α-synuclein fibril-destabilizing properties had been shown before [53]. This effect of SJ extract could be probably due to the activation of the SKN-1 pathway, yet involvement of other signaling pathways could be possible. A former study also revealed the effect of certain phytochemicals from *Dioscorea alata* L. tubers on reducing α-synuclein aggregates that was linked to enhanced SKN-1 nuclear localization [54]. Consistently, rutin—an antioxidant—was also able to reduce aggregate formation.

To study the effect of the extract on counteracting formation and toxicity of Aβ, a paralysis assay was performed using a mutant nematode strain expressing human Aβ peptides in the muscles. Upon increasing the temperature, a significant delay of onset of paralysis compared to control was observed in SJ treated groups indicating the protective properties of the extract towards Aβ toxicity. Notably, rutin treatment did not significantly delay onset of paralysis, indicating that SJ components can target distinct pathways that are not sensitive to rutin. Apigenin might be such a component of SJ extract. Indeed, its neurotrophic and anti-amyloidogenic activities together with a decrease in the levels of insoluble Aβ have been previously demonstrated in a mouse model of Alzheimer’s disease [55]. SKN-1 pathway activation can be contributing to the observed effect as its orthologue, Nrf2, activation was previously responsible for exerting neuroprotection against Aβ in mouse model but contribution of other pathways cannot be excluded from our experiments [56].

The *C. elegans* model is a cost-effective approach for the screening of neuroactive compounds using a variety of biochemical assays. Owing to the simplicity of the model organism, not all aspects of immune and inflammatory responses can be assessed [57]. Therefore, complementary studies are required to explore the specific molecular mechanism of action of SJ extract, identify the active principles and test their efficacy and safety in more complex models, such as mammalian organisms. Isolation of the active principles and detailed analysis of the targeted physiological pathways are required. Moreover, for further confirmation of the SKN-1 activity in SJ extract, expression of other antioxidant genes like *gcs-1*, which is downstream to SKN-1 could be measured in future studies. Also the activity of SJ extract on the expression of *gst-4* after exposure to mild oxidative stress conditions could be also assayed as the antioxidant activity of SJ could probably be more pronounced after exposure to stressors. However, our findings clearly identify SJ extract and its components as a source of promising novel neuroprotective compounds. In addition, this is the first study to report that SJ extract shows great potential towards modulating protein homeostasis and decreasing the development of age-related diseases such as HD, PD, and AD.

## Conclusions

This study confirms the mediation of stress resistance and neuroprotection by a *Styphnolobium japonicum* extract. Use of the extract can help towards a healthy process of aging and offer protection from age-associated disorders. The observed oxidative stress resistance effect, manifested by increasing survival rate in juglone treated *daf-16* mutant worms, is possibly achieved through promoting nuclear localization of SKN-1/Nrf2 transcription factor. Modulation of expression of the downstream *gst-4* gene confirms the role of the SKN-1 pathway in the observed beneficial effects of SJ extract.

### Supplementary Information

**Additional file 1:** **Supplementary Figure S1.** Representative fluorescence images of Cl2166 *C. elegans* worms showing *gst-4*::GFP expression.

**Additional file 2:** **Supplementary Figure S2.** Representative fluorescence images of AM141 *C. elegans* worms showing polyQ40::YFP expression.

## Data Availability

Data and materials will be made available to researchers upon reasonable request. Contact Dr. Sara Thabit: sara.thabit@guc.edu.eg.

## Abbreviations

ASH: Amphid sensilla
Aβ: Amyloid β
AD: Alzheimer’s disease
BHA: Butylated hydroxyl anisole
*C. elegans*: *Caenorhabditis elegans*
Cu^+^: Cuprous
Cu^2+^: Cupric
CUPRAC: Cupric ion reducing antioxidant capacity
EGCG: Epigallocatechin gallate
*E. coli*: *Escherichia coli*
GST-4: Glutathione S-transferase-4
HD: Huntington’s disease
PD: Parkinson’s disease
PolyQ40: Polyglutamine
*S. japonicum*: *Styphnolobium japonicum*
ROS: Reactive oxygen species
SOD-3: Superoxide dismutase-3
