# Supplementary material for: A fruit extract of Styphnolobium japonicum (L.) counteracts oxidative stress and mediates neuroprotection in Caenorhabditis elegans
Source: BMC Complement Med Ther. 2023 Sep 19;23:330. doi: 10.1186/s12906-023-04149-8 (PMC10507854; doi:10.1186/s12906-023-04149-8)
Supplement: Supplementary file 1 — Additional file 1: Supplementary Figure S1. Representative fluorescence images of Cl2166 C. elegans worms showing gst-4::GFP expression. [file 12906_2023_4149_MOESM1_ESM.pdf]

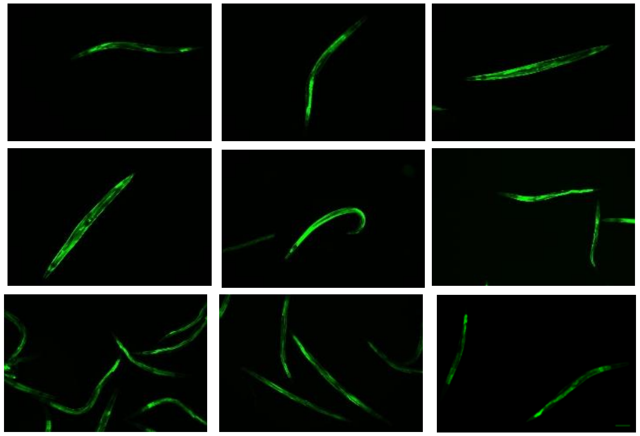

(a) Untreated Control

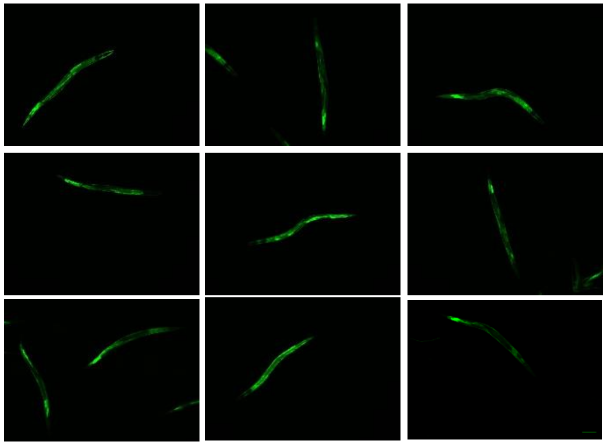

(b) Solvent Control

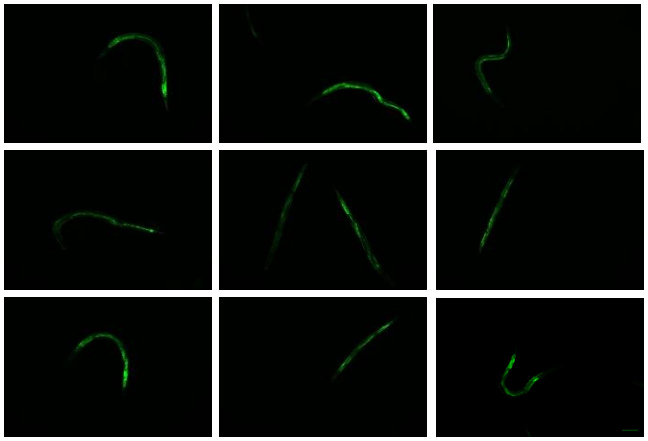

(c) SJ 100 µg/ml

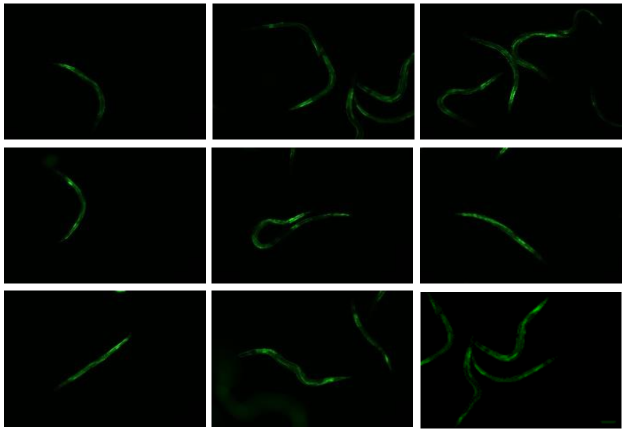

(d) SJ 200 µg/ml

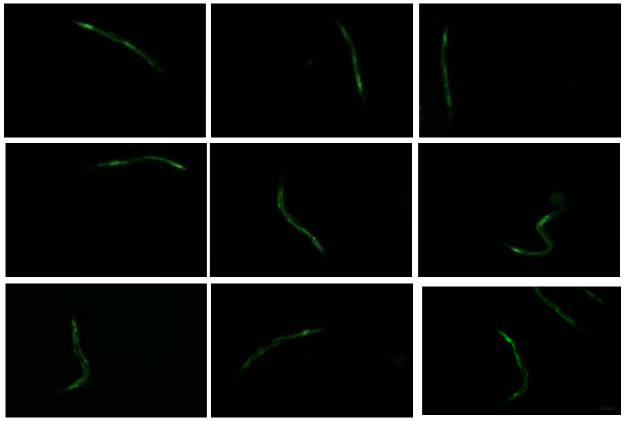

(e) SJ 300 µg/ml

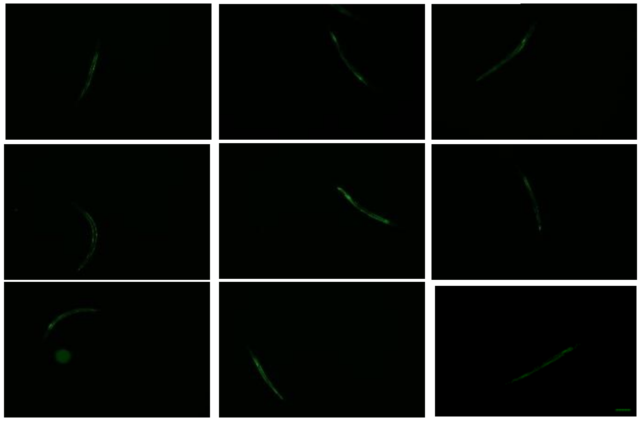

(f) EGCG 50 µg/ml

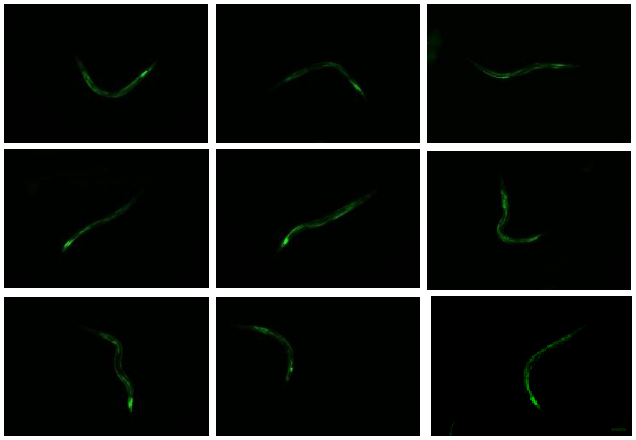

(g) Rutin 100 µg/ml

Supplementary Figure S1: Representative images of worms for GST-4 expression, scale bar = 100 µm.
